# Detection of Antibiotic Resistant *Staphylococcus* *aureus* from Milk: A Public Health Implication

**DOI:** 10.3390/ijerph120910254

**Published:** 2015-08-25

**Authors:** Muyiwa Ajoke Akindolire, Olubukola Oluranti Babalola, Collins Njie Ateba

**Affiliations:** 1 Department of Biological Sciences, School of Environmental and Health Sciences, Faculty of Agriculture, Science and Technology, North-West University, Mmabatho, Mafikeng Campus, South Africa; E-Mails: bonajoke@yahoo.com (M.A.A.); olubukola.babalola@nwu.ac.za (O.O.B.); 2 Food Security and Safety Niche Area, Faculty of Agriculture, Science and Technology, North-West University, Mmabatho, Mafikeng 2735, South Africa

**Keywords:** *Staphylococcus aureus*, MALDI-TOF mass spectrometry, methicillin resistance, *nuc* specific PCR, virulence genes

## Abstract

The aim of this study was to investigate the occurrence, antibiotic susceptibility profiles, and virulence genes determinants of *S. aureus* isolated from milk obtained from retail outlets of the North-West Province, South Africa. To achieve this, 200 samples of raw, bulk and pasteurised milk were obtained randomly from supermarkets, shops and some farms in the North-West Province between May 2012 and April 2013. *S. aureus* was isolated and positively identified using morphological (Gram staining), biochemical (DNase, catalase, haemolysis and rapid slide agglutination) tests, protein profile analysis (MALDI-TOF mass spectrometry) and molecular (*nuc* specific PCR) methods. The antimicrobial resistance profiles of the isolates were determined using the phenotypic agar diffusion method. Genes encoding enterotoxins, exfoliative toxins and collagen adhesins were also screened using PCR. Among all the samples examined, 30 of 40 raw milk samples (75%), 25 of 85 bulk milk samples (29%) and 10 of 75 pasteurised milk samples (13%) were positive for *S. aureus*. One hundred and fifty-six PCR-confirmed *S. aureus* isolates were obtained from 75 contaminated milk samples. A large proportion (60%–100%) of the isolates was resistant to penicillin G, ampicillin, oxacillin, vancomycin, teicoplanin and erythromycin. On the contrary, low level resistance (8.3%–40%) was observed for gentamicin, kanamycin and sulphamethoxazole. Methicillin resistance was detected in 59% of the multidrug resistant isolates and this was a cause for concern. However, only a small proportion (20.6%) of these isolates possessed PBP2a which codes for Methicillin resistance in *S. aureus*. In addition, 32.7% of isolates possessed the *sec* gene whereas the *sea*, *seb sed*, *see, cna*, *eta*, *etb* genes were not detected. The findings of this study showed that raw, bulk and pasteurised milk in the North-West Province is contaminated with toxigenic and multi-drug resistant *S. aureus* strains. There is a need to implement appropriate control measures to reduce contamination as well as the spread of virulent *S. aureus* strains and the burden of disease in humans.

## 1. Introduction

Milk is high in nutrients such as vitamins, proteins, lactose, fat, minerals and water. It plays an important role in assisting individuals meet their nutrient requirements [1,2,3,4]. It has been reported worldwide that foods of animal origin, particularly milk and other dairy products, are often associated with food-borne diseases if proper sanitary and health care procedures are not implemented during the production and marketing of these products [5,6,7]. This is mainly due to the fact that milk may serve as an excellent medium for the survival and growth of many different types of pathogenic microorganisms. Milk is regarded as a potential vehicle for the transmission of bacteria, including staphylococci to humans [8,9].

Staphylococci are bacteria that easily grow and establish themselves as commensals on the skin and mucous membranes of warm-blooded animals [10,11,12,13,14]. In line with this, a number of coagulase positive and coagulase negative species have been isolated from humans and animals [11,12,15,16,17]. However, *S. aureus* is well known as a pathogen in both human and animal medicine, and it is currently considered as one of the world’s most important pathogens [18]. *S. aureus* is known to cause a number of pathological conditions in humans and animals that range from mild skin infections, bacteremia, systemic diseases, osteomyelitis to the more complicated toxic shock syndrome and staphylococcal food poisoning (SFP) [18,19,20].

*S. aureus* is reported to be one of the most common causative agents of food poisoning associated with the consumption of raw milk and milk products [21]. Foodstuff contamination may occur directly from infected food-producing animals or may result from poor hygiene during production processes, or the retail and storage of food, since humans may also harbour microorganisms [8,9,22]. As such, food products such as milk, cheese, yoghurt and other dairy products have been implicated as potential sources for the transmission of the pathogen to humans [8]. Moreover, foods contaminated with antibiotic resistant bacteria represent ideal vehicles for the transmission of antibiotic resistant strains [23,24].

Antimicrobial resistance is an important health problem worldwide [25,26]. The development of resistance, both in human and animal bacterial pathogens, has been ascribed to the extensive therapeutic use of antimicrobials or with their use as growth promoters in animal feed production [27,28,29,30]. Methicillin-resistant *S. aureus* (MRSA) was first described in 1961, shortly after the introduction of methicillin [31]. *S. aureus* becomes methicillin resistant by acquisition of the *mecA* gene which encodes a modified penicillin binding protein (PBP2a) that has a low affinity for β-lactams [8,32,33]. The modified PBP2a in MRSA isolates is therefore capable of replacing the biosynthetic functions of normal penicillin binding proteins even in the presence of β-lactam antibiotics, thereby preventing cell lysis. As such, *S. aureus* strains producing PBP2a are resistant to all β-lactam antibiotics [32] as well as other classes of antibiotics. Since the development of MRSA, vancomycin has been used as the antibiotic of choice to treat infections caused by *S. aureus* strains that are resistant to methicillin and oxacillin. In addition, the emergence of vancomycin-resistant *S. aureus* has been reported in some studies [16,34,35].

The multi-drug resistant *S. aureus* strains may have an increased ability to spread, especially if they are also enhanced with virulence genes. This does not only provide therapeutic challenges for clinicians but may be very detrimental to human health [36]. The study was designed to determine the occurrence of *S. aureus* and MRSA strains in milk products obtained from some supermarkets, shops and farms in the North West Province of South Africa. Moreover, the possible health risks to consumers based on the presence of virulence genes and antibiotic resistance profiles of the isolates were also investigated.

## 2. Experimental Section

### 2.1. Study Design

A cross-sectional study design was used to determine the bacteriological analysis of raw, tank and pasteurised milk in the North-West Province of South Africa.

### 2.2. Study Site

This study was conducted in Mafikeng town, North-West Province of South Africa. The province has an estimated population of 3,509,953 million inhabitants [37]. The North-West province is located in the north of South Africa, on the Botswana border. It is surrounded by the Kalahari desert to the west, Gauteng province to the east, and the Free State to the south.

### 2.3. Sample Collection

From May 2012 to April 2013, a total of 200 milk samples including raw, bulk and pasteurised milk were randomly collected from 18 sources (supermarkets, shops and farms) in the four districts of the North-West province of South Africa (Table 2). Two to five (the number depends on the size of the district) representative stores were chosen in each district for sampling. Each milk sample (500 mL) was collected, labelled properly, kept at 4 °C in an insulated ice box and transported to the laboratory for analysis. Upon arrival in the laboratory, samples were analysed for *S. aureus* within 4 h of collection.

### 2.4. Enrichments and Isolation of S. aureus

Isolation of *S. aureus* was done as described [38]. Isolation involved a non-selective enrichment procedure [39] where a 5 mL aliquot of each milk sample was mixed with 5 mL double strength Trypticase Soy Broth (TSB) (Biolab, Wadeville, South Africa). This preparation was incubated for 3 h at 37 °C. Ten milliliters of a single-strength TSB supplemented with 20% NaCl was later added and incubated for 24 h at 37 °C. Aliquots of 0.1 mL were spread-plated onto mannitol salt agar (MSA) (Biolab, Modderfontein, South Africa). The plates were incubated aerobically at 37 °C for 24 h. Presumptive *S*. *aureus* colonies that were yellow in colour from each MSA (Biolab, Modderfontein, South Africa) plate were sub-cultured onto fresh MSA (Biolab, Modderfontein, South Africa) plates for isolate purification and incubated aerobically at 37 °C for 24 h. Isolates were retained for further identification.

### 2.5. Bacterial Identification

Presumptive *S. aureus* colonies were identified by standard microbiological tests which included Gram-staining; catalase testing (using 3% hydrogen peroxide); slide agglutination test; DNase test; and the oxidation and fermentation of mannitol salt agar (Biolab, Modderfontein, South Africa) [40].

#### 2.5.1. Biochemical Identification of Staphylococci

Isolates were Gram-stained using standard techniques [41]. Colonies that were Gram-positive cocci, arranged in clusters, were further identified. *S. aureus* isolates were screened for DNase production using the DNase agar (Fluka^®^ Analytical: Sigma-Aldrich Chemie GmbH, Buchs, Switzerland) [42], the catalase test [43], haemolysin test using 5% (w/v) sheep blood agar [44] and slide agglutination test using the PASTOREX^TM^ STAPH-PLUS (Bio-Rad Laboratories Diagnostics Group, Redmond, WA, USA) which facilitates simultaneous detection of bound coagulase also known as clumping factor; protein A; and capsular polysaccharides that are specific for *S*. *aureus*. All tests were performed according to standard guidelines and *S. aureus* (ATCC^®^ 25923) was used as a positive control in each test protocol.

### 2.6. Determination of the Identities of Isolates using the Matrix-Assisted Laser Desorption/Ionization Mass Spectrometry (MALDI-TOF MS)

Pure isolates were sub-cultured on MSA agar and plates incubated at 37 °C for 24 h. The plates were transported to the Microbiology Laboratory of the University of Pretoria (South Africa) for MALDI-TOF MS analysis by the equipment specialist using standard protocols and procedures. The MALDI Biotyper identifies organisms using the Matrix-Assisted Laser Desorption/Ionization Mass Spectrometry by measuring the unique protein fingerprint of highly abundant proteins in an organism. The characteristic patterns of these proteins are used to reliably identify the particular microorganism by matching the respective patterns with an extensive open database. Analysis was performed and isolates identified to species level.

### 2.7. Molecular Identification by PCR

#### 2.7.1. DNA Extraction

Genomic DNA was extracted using a ZR Genomic DNA^TM^–Tissue MiniPrep kit (Zymo Research Corp, Irvine, CA, USA). The extraction protocol used was as instructed by the manufacturer. The quality and quantity of the extracted genomic DNA was determined using a Nanodrop 2000 Spectrophotometer (Thermo Scientific, Waltham, MA, USA) and diluted to a working concentration of 50 ng/μL. The eluted DNA was stored at −20 °C and used for molecular identification of the isolates.

#### 2.7.2. 16S rRNA Specific PCR for the Detection of Staphylococci

The identities of organisms belonging to the genus staphylococci were determined using a 16S rRNA specific PCR assay that amplifies the 16S rRNA gene sequence specific for staphylococci [45]. Primer sequences used are shown in Table 1. PCR was conducted using a C1000 Touch Thermal Cycler (Bio-Rad, Johannesburg, South Africa) and the cycling conditions were an initial denaturation at 94 °C for 5 min; followed by 30 cycles of 94 °C for 30 seconds, 64 °C for 30 seconds, 72 °C for 60 seconds and a final elongation step of 72 °C for 5 min. The PCR products were stored at 4 °C and later separated by agarose gel electrophoresis.

#### 2.7.3. Specific PCR for the Identification of *Staphylococcus aureus*

The identities of *S*. *aureus* isolates were confirmed using a specific PCR that targeted the thermonuclease (*nuc*) gene specific for *S*. *aureus* [46] using primers sequences listed in Table 1. PCR was conducted using a C1000 Touch Thermal Cycler (Bio-Rad, Johannesburg, South Africa) and the cycling conditions utilized were initial denaturation of 94 °C for 5 min; followed by 30 cycles of 94 °C for 30 seconds, 55 °C for 30 seconds, 72 °C for 60 seconds and a final elongation step of 72 °C for 5 min. The PCR products were stored at 4 ^o^C and later separated by agarose gel electrophoresis. After identification, each *S. aureus* strain was stored at −80 °C in Brain Heart Infusion broth (BHI) (Oxoid, Basingstoke, UK) with glycerol (15% v/v).

### 2.8. Multiplex Polymerase Chain Reaction (PCR) Detection of Gene Sequences that Encode Virulence Determinants in *S. aureus*

Multiplex PCR was used to screen for the presence of genes encoding different virulent determinants: staphylococcal enterotoxins A to E (*sea*, *seb*, *sec*, *sed* and *see*) [47], exfoliative toxins A and B (*eta* and *etb*) [48] and cna gene [49]. Primer sequences used for the PCR assays are listed in Table 1. Two sets of multiplex PCR mixes containing enterotoxins A to E primers and another with *eta*, *etb* and *cna* primers were prepared. Each of the multiplex PCR mix contained 10–50 ng of template DNA, 1.5 µL of 25 mM MgCl_2_ (Promega Corporation, Madison, USA), 1.5 µL of 5 × Green Go Taq® reaction buffer (Promega Corporation, Madison, WI, USA), 12.5 µL of 2 × PCR master mix (Thermo Scientific, Johannesburg, Sounth Africa) and 50 pmol of each primer (Inqaba Biotech, Pretoria, Sounth Africa). The volume of this reaction mixes was adjusted to 25 µL with nuclease free water. DNA amplification was carried out in a C1000 Touch Thermal Cycler (Bio-Rad, Johannesburg, South Africa) with the following cycling conditions: an initial denaturation of 94 °C for 5 min was followed by 35 cycles of amplification (denaturation at 94 °C for 2 min, annealing at 57 °C for 2 min and extension at 72 °C for 1 min), ending with a final extension at 72 °C for 7 min.

### 2.9. Agarose Gel Electrophoresis of DNA Extracted and PCR Products

DNA extracted and PCR products were separated by electrophoresis on a 0.8% (w/v) and 2% (w/v) agarose (Sigma-Aldrich, Seakme®, Rockland, ME, USA) gel. A 100 bp or 1 kb DNA ladder (Fermentas, Glen Burnie, GB, USA) was included in all PCR and fingerprinting gels as a molecular weight standard. ChemiDoc^TM^ MP Imaging system (Bio-Rad, Hercules, CA, USA) was used to visualise and capture the images.

**Table 1:** Oligonucleotide primers used for molecular identification and multiplex detection of virulence determinant genes.

Primer	Sequence	Target Gene	Amplicon Size (bp)
16S rRNA F	GTAGGTGGCAAGCGTTACC	*16S rRNA*	228
16S rRNA R	CGCACATCAGCGTCAG		
Nuc F	GCGATTGATGGTGGATACGGT	*Nuc*	279
Nuc R	AGCCAAGCCTTGACGAACTAAAGC		
Sea F	GGTTATCAATGTGCGGGTGG	*Sea*	102
Sea R	CGGCACTTTTTTCTCTTCGG		
Seb F	GTATGGTGGTGTAACTGAGC	*Seb*	164
Seb R	CCAAATAGTGACGAGTTAGG		
Sec F	AGATGAAGTAGTTGATGTGTATGG	*Sec*	451
Sec R	CACACTTTTAGAATCAACCG		
Sed F	CCAATAATAGGAGAAAATAAAAG	*Sed*	278
Sed R	ATTGGTATTTTTTTTCGTTC		
See F	AGGTTTTTTCACAGGTCATCC	*See*	209
See R	CTTTTTTTTCTTCGGTCAATC		
Eta F	ATATCAACGTGAGGGCTCTAGTAC	*Sta*	1155
Eta R	ATGCAGTCAGCTTCTTACTGCTA		
Etb F	CACACATTACGGATAATGCAAG	*Etb*	604
Etb R	TCAACCGAATAGAGTGAACTTATCT		
Cna F	AAAGCGTTGCCTAGTGGAGA	*Can*	192
Cna R	AGTGCCTTCCCAAACCTTTT		

### 2.10. Determination of the Antibiotic Resistance Profiles of S. aureus Isolates

#### Antibiotic Disc Susceptibility Test, Detection of Multiple Antibiotic Resistant (MAR) Phenotypes and Penicillin Binding Protein (PBP2a)

An antibiotic susceptibility test was performed using the Kirby-Bauer disc diffusion method [50]. Susceptibilities of the isolates to a panel of 11 different antimicrobial agents that include penicillin G (10 μg), oxacillin (5 μg), ampicillin (10 μg), streptomycin (10 μg), kanamycin (30 μg), gentamicin (10 μg), erythromycin (15 μg), oxytetracycline (30 μg), vancomycin (30 μg), teicoplanin (30 μg) and sulphamethoxazole (15 μg) (Mast Diagnostics, Merseyside, UK) were determined. The test was performed and results were interpreted according to standard guidelines [51]. All isolates were tested for the production of PBP2a, the protein encoded by *mecA*, using the PBP2a latex agglutination assay (Bio-Lab, Pretoria, South Africa) that employs a serological procedure for the detection of Methicillin and Oxacillin resistant *S. aureus*. The test was performed as instructed by the manufacturer. The MAR phenotypes were generated for isolates resistant to three and more antibiotics using the abbreviations that appear on antibiotic discs [52]. A methicillin susceptible *S. aureus* (ATCC^®^ 25923) and an MRSA strain (ATCC^®^ 43300) were used as a negative and positive control, respectively.

## 3. Results

### 3.1. Presumptive Detection of Staphylococcus Species in Milk Samples Based on Cultural Characteristics

A total of 200 milk samples were collected and screened for the presence of *Staphylococcus* species. This comprised of 40 samples from raw milk, 85 from tank milk and 75 from pasteurised milk. The bacterium was detected in 75% (30/40), 29% (25/85), 13% (10/75) of raw milk (RM), tank milk (TM) and pasteurised milk (PM), respectively. The overall occurrence of *S. aureus* contamination in the milk samples was 32.5% (65/200). 

A total of 380 isolates obtained from the contaminated milk samples were screened for the characteristics of *S. aureus*. Only isolates that satisfied both preliminary and confirmatory biochemical tests, such as Gram staining, catalase test, slide agglutination test, DNase test were considered. A large proportion 56% (211/380) of the isolates were positively identified by using the slide agglutination test. All the isolates were Gram-positive and catalase-positive and a large number 65% (138/211) of the isolates were positive for DNase production. The majority of the isolates 74% (156/211) showed haemolytic activity on sheep blood agar plates: 50% (78/156) of the haemolytic *S aureus* isolates produced α-haemolysin while 44% (69/156) displayed β-haemolysis and 6% (9/156) were α and β-haemolytic. The results revealed that *S. aureus* was frequently isolated from milk samples obtained from the North-West Province. Although all the milk samples were contaminated with *S. aureus*, isolates obtained from Coligny, Wolmaranstad, Lehurutshe and Madibogo were not positively identified by the slide agglutination test and thus not included in the study. The results in Table 5A and Table 5B indicate the number of isolates screened from the different sampling areas and those that were positive for the different tests. All the isolates that were positively identified as *S. aureus* by the slide agglutination test were subjected to confirmatory identification using molecular methods. In this study, staphylococci were detected in all the different types of milk samples analysed. Bacteria were more frequently isolated from raw milk than tank milk and pasteurised milk obtained from the supermarkets.

### 3.2. Molecular Characterisation of S. aureus Isolates from Milk

#### 3.2.1. Specific PCR for the Identification of *Staphylococcus aureus*

A total of 211 *S. aureus* isolates that were positively identified by the Staph Xtra Latex agglutination test were subjected to specific simplex PCR analysis for the detection of 16S rRNA gene and the *S. aureus* thermonuclease (*nuc*) gene. The 16S rRNA gene is specific for the genus *Staphylococcus* while the *nuc* gene is species specific for *S. aureus*. A Figure of a 2% (w/v) agarose gel depicting the 16S rRNA gene fragments amplified by PCR using genomic DNA extracted from *S. aureus* isolates was reported as Supplementary Material. The 16S rRNA and *nuc* gene fragments with the expected amplicon sizes of 228bp and 279bp were respectively obtained and the agarose gel pictures were reported as Supplementary Material. Table 2 shows the number of isolates screened from the different sampling sites and those that were positive for the targeted genes.

As shown in Table 2, the proportion of isolates that was positive for the 16S rRNA gene (100%) PCR analysis was higher than those that possessed the *nuc* gene (74%). This is not surprising since the *nuc* gene is more specific in detecting isolates belonging to the species *S. aureus* compared to the 16S rRNA gene that is common to the genus *Staphylococcus* and will detect other *Staphylococcus* species in addition to *S. aureus.*

#### 3.2.2. Detection of Virulence Genes in *Staphylococcus aureus* Isolates

A total of 156 *S. aureus* isolates were subjected to multiplex PCR analysis for the detection of virulence genes. Results obtained are shown in Table 2. Only the *sec* virulence gene was amplified from isolates screened and the *sea*, *seb*, *sed*, *see*, *cna*, *eta* and *etb* genes were not detected in any of the isolates. Generally, 51 (32.7%) of the isolates possessed the *sec* gene and a large proportion of the isolates were obtained from Rooigrond (47%) and Disaneng (24%). None of the isolates from Rustenburg, Mabule, Taung and Lehurutshe possessed the *sec* virulence determinant. A 2% (w/v) agarose gel depicting the *sec* virulence gene fragments amplified by PCR using genomic DNA extracted from *S. aureus* isolates was reported as Supplementary Material. Amplicons obtained were of the expected sizes (451 bp).

**Table 2:** Areas where milk samples were collected and number of *S. aureus* isolates positive for the 16S rRNA, *nuc* and virulence gene determinants.

Sample Source		Number of Isolates Tested	Number of Isolates Positive for the Targeted Genes									
			*16S rRNA* gene	*nuc* gene	*sea* gene	*seb* gene	*sec* gene	*see* gene	*sed* gene	*cna* gene	*eta* gene	*etb* gene
Coligny	10											
Carletonville	10	16	16	12	0	0	2	0	0	0	0	0
Potchefstroom	10	8	8	6	0	0	1	0	0	0	0	0
Wolmaranstad	10											
Zeerust	10	12	12	10	0	0	2	0	0	0	0	0
Swartruggens	10	11	11	5	0	0	1	0	0	0	0	0
Rustenburg	10	18	18	15	0	0	0	0	0	0	0	0
Rooigrond	15(15)*	34	34	30	0	0	24	0	0	0	0	0
Mabule	10	10	10	5	0	0	0	0	0	0	0	0
Mafikeng	15(15)*	15	15	12	0	0	4	0	0	0	0	0
Disaneng	20(15)*	30	30	24	0	0	12	0	0	0	0	0
Taung	10	6	6	5	0	0	0	0	0	0	0	0
Vryburg	10	20	20	14	0	0	2	0	0	0	0	0
Setlagole	10	12	12	8	0	0	1	0	0	0	0	0
Stella	10	12	12	8	0	0	1	0	0	0	0	0
Madibogo	10											
Lehurutshe	10	5	5	0	0	0	0	0	0	0	0	0
Tshidilamolomo	10	2	2	2	0	0	1	0	0	0	0	0
Total		211	211	156			51					

* Number of raw milk samples collected from the different areas based on availability.

### 3.3. Identification of S. aureus Isolates Using the MALDI-TOF Mass Spectrometry

A total of 36 *S. aureus* isolates that were positive for the 16S rRNA gene and the *nuc* gene PCR analysis were subjected to the MALDI Biotyper identification tool using the Matrix-Assisted Laser Desorption/Ionization Mass Spectrometry. Using the isolates obtained from the present study as unknowns, highly reproducible mass spectral profiles were obtained and compared with those of the reference spectra deposited in an extensive open database. Unique protein fingerprints of highly abundant proteins in each isolate were obtained. A representation of the proteins profiles, peaks and mass spectral profiles of representative *S. aureus* isolates from milk obtained from Rooigrond was reported as Supplementary Material. The identities of isolates obtained after analysis and the scores used to determine the reliability of the technique in classifying an isolate as member of a particular genus and species are shown in Table 3. All the isolates were positively identified as *S. aureus* to species level.

**Table 3:** Number of *S. aureus* isolates positively identified using the MALDI Biotyper technique.

Isolate Number	Score Value	Identity Based on MALDI-TOF MS Analysis
		Best Match
1RO3	2.234	*Staphylococcus aureus*
1RO4	2.097	*Staphylococcus aureus*
1RO5	2.223	*Staphylococcus aureus*
1RO6	2.068	*Staphylococcus aureus*
1RO9	2.076	*Staphylococcus aureus*
2RO1	2.234	*Staphylococcus aureus*
2RO2	2.259	*Staphylococcus aureus*
2RO4	2.124	*Staphylococcus aureus*
2RO5	2.232	*Staphylococcus aureus*
3RO1	2.177	*Staphylococcus aureus*
3RO2	2.31	*Staphylococcus aureus*
3RO3	2.255	*Staphylococcus aureus*
3RO6	2.048	*Staphylococcus aureus*
3RO9	1.921	*Staphylococcus aureus*
4R1	2.288	*Staphylococcus aureus*
4R2	2.338	*Staphylococcus aureus*
4R3	2.224	*Staphylococcus aureus*
4R4	2.249	*Staphylococcus aureus*
4R5	2.296	*Staphylococcus aureus*
14Z1	2.228	*Staphylococcus aureus*
15Z1	2.188	*Staphylococcus aureus*
16Z1	2.086	*Staphylococcus aureus*
16Z2	2.254	*Staphylococcus aureus*
20Z1	2.129	*Staphylococcus aureus*
20Z2	2.127	*Staphylococcus aureus*
20Z3	2.179	*Staphylococcus aureus*
20Z4	2.109	*Staphylococcus aureus*
21Z1	2.215	*Staphylococcus aureus*
21Z2	2.291	*Staphylococcus aureus*
22Z1	2.165	*Staphylococcus aureus*
22Z2	2.113	*Staphylococcus aureus*
22Z3	2.163	*Staphylococcus aureus*
22Z4	2.069	*Staphylococcus aureus*
6S3	2.027	*Staphylococcus aureus*
6S1	2.129	*Staphylococcus aureus*
7S1	2.194	*Staphylococcus aureus*

As shown in Table 3, the score value for best match organism of all the isolates screened ranged from 2.027 to 2.338 indicating a reliable genus and species identification. Moreover, the greatest peak density or hits of mass ions were detected at about *m/z* 2800 and all the isolates correctly identified to genus and species level as *S. aureus*. Generally, all the isolates showed similar profiles and it is suggested that the consistent mass ions at *m/z* 2000 to *m/z* 10000 appear to be biomarkers specific for the *S. aureus* specie. When the *nuc* specific PCR and the MALDI-TOF MS data were compared, it was evident that both could be used for correct identification of bacteria isolates under controlled laboratory and experimental conditions.

### 3.4. Antibiotic Resistance of S. aureus Isolates from Milk Samples

A total 156 *S. aureus* isolates from milk obtained from different sampling sites in the North-West Province that possessed the *nuc* gene were tested to evaluate their susceptibilities against a panel of 11 antimicrobial agents. Data depicting the susceptibilities of the isolates were presented in percentages as shown in Table 4. A large proportion (60%–100%) of the *S. aureus* isolates obtained from Carletonville, Potchefstroom and Zeerust were resistant to penicillin G, ampicillin, oxacillin, streptomycin, vancomycin, and erythromycin. In addition, a similarly large proportion of isolates from Swartruggens, Rustenburg, Stella, Setlagole, Vryburg, Taung and Disaneng were resistant to penicillin G and oxacillin (Table 4). Despite the fact that a relatively large proportion (50%–71%) of the isolates from Vryburg, Setlagole and Disaneng were resistant to gentamicin, on the contrary, only a small proportion (8.3%–40%) of isolates from the other stations sampled were resistant to this antimicrobial agent. With the exception of Disaneng, Carletonville and Potchefstroom all isolates obtained from the other sample sites were highly susceptible to kanamycin. Similar low level resistance was observed against sulphamethoxazole (Table 4). A cause for concern is the fact that multiple antibiotic resistant (MAR) isolates that are also resistant to oxacillin were detected in this study. These isolates could serve as reservoirs for the transmission and spread of antibiotic resistant determinants within a population.

### 3.5. Latex Agglutination Assay for the Detection of Penicillin Binding Protein 2a (PBP2a) in S. aureus Isolates from Milk Samples

A total of 92 MAR antibiotic resistant *S. aureus* strains isolated from milk obtained from different sampling sites in the North-West Province were subjected to a serological test using the Latex PBP2a test kit. This aim was to detect PBP2a which is used to identify Methicillin resistance in *S. aureus* and the Latex technology provides an alternative to the PCR technique to detect the *mecA* gene product serologically. All the 92 isolates were resistant to oxacillin based on the antibiotic disc susceptibility test. Results obtained are shown in Table 5. Despite the fact that only a small proportion (19) 20.6% of the isolates were positively identified to possess the gene responsible for Methicillin resistance in *S. aureus*, the presence of this gene was a cause for concern. Moreover, the ability to determine whether a test is positive or not depends on the person interpreting the results and therefore the actual occurrence of PBP2a may be higher than the reported value. Consequently, the isolates were also screened by PCR analysis for the *mecA* gene.

**Table 4:** The number and percentages of *S. aureus* isolates from various sampling sites resistant to different antibiotics.

Sampling Area		PG	AMP	OX	S	K	GM	VA	TEC	TE	E	SMX
Carletonville NT = 12	NR	12	12	12	7	6	1	10	3	8	12	0
	%	100	100	100	58	50	8.3	83	25	66.7	100	0
Potchefstroom NT = 6	NR	6	6	6	4	3	1	6	6	0	6	1
	%	100	100	100	67	50	17	100	100	0	100	17
Zeerust NT = 10	NR	10	10	9	7	4	3	9	10	2	10	1
	%	100	100	90	70	40	30	90	100	20	10	10
Swartruggens NT =5	NR	5	4	3	5	1	0	4	4	3	5	1
	%	100	80	60	100	20	0	80	80	60	100	20
Rustenburg NT = 15	NR	15	15	11	8	3	5	15	15	9	9	3
	%	100	100	73	53	20	33	100	100	60	60	20
Stella NT = 8	NR	8	3	4	4	0	2	5	7	7	3	3
	%	100	38	50	50	0	25	63	88	88	38	38
Setlagole NT = 8	NR	8	4	7	3	2	5	5	5	8	6	6
	%	100	50	88	38	25	63	63	63	100	75	75
Vryburg NT = 14	NR	14	6	10	8	6	10	9	9	13	7	8
	%	100	43	71	57	43	71	64	64	93	50	57
Taung NT = 5	NR	5	0	1	0	0	2	2	5	5	0	0
	%	100	0	20	0	0	40	40	40	40	0	0
Disaneng NT = 24	NR	17	18	23	19	17	14	11	12	11	13	4
	%	71	75	96	79	71	58	46	50	46	54	17
Mafikeng NT = 11	NR	9	9	1	8	5	1	7	11	8	11	4
	%	82	82	9	73	45	9	64	100	73	100	36
Mabule NT = 5	NR	4	3	1	4	0	0	2	4	4	5	1
	%	80	60	20	80	0	0	40	80	80	100	20
Rooigroond NT = 30	NR	9	9	5	9	8	3	24	25	19	19	3
	%	30	30	17	30	27	10	80	83	63	63	10
Tshidilamolomo NT = 2	NR	2	1	0	1	0	0	1	0	0	1	0
	%	100	50	0	50	0	0	50	0	0	50	0

PG = penicillin G; Amp = ampicillin; OX = oxacillin; S = streptomycin; K = kanamycin; GM = gentamicin; VA = vancomycin; TEC = teicoplanin, TE=tetracycline, E = erythromycin, SMX = sulphamethoxazole. NT = Number tested; NR = number resistant.

### 3.6. Multiple Antibiotic Resistance Phenotypes of S. aureus Isolates from Milk Samples

Multiple antibiotic resistance (MAR) phenotypes were generated from 110 *S. aureus* isolates showing resistance to three or more antibiotics [52]. Data indicating the predominant MAR phenotypes are shown in Table 6. The MAR phenotype PG-AMP-VA-TEC-TE-E was observed in 60% of isolates from Taung, while 25% of those from Mafikeng displayed this phenotype. The MAR phenotypes PG-AMP-OX-S-VA-TEC-E was dominant among 50%, 30% and 29% of samples from Potchefstroom, Zeerust and Carletonville, respectively. The predominant MAR phenotypes for isolates from raw milk from Disaneng and pasteurized milk from Vryburg were PG-AMP-OX-S-K-CN-VA-TEC-E and PG-AMP-SK-VA-TEC-TE-E-SMX obtained at 20% and 30%, respectively. These results reveal that MAR *S. aureu*s were isolated from milk samples. It is therefore suggested that these MAR isolates may have severe health implications in individuals who consume such milk products.

**Table 5:** Proportion of *S. aureus* isolates from various sampling sites that were positive for the PBP2a Latex agglutination test.

Sample Source	Number of Isolates Tested	Number of Isolates Positive for the PBP2’ Test
Carletonville	12	3
Potchefstroom	5	2
Zeerust	9	2
Swartruggens	3	1
Rustenburg	12	2
Rooigrond	5	0
Mafikeng	4	0
Vryburg	7	1
Setlagole	5	0
Stella	5	3
Mabule	3	0
Disaneng	22	5
Total	92	19

**Table 6:** Predominant multiple antibiotic resistance (MAR) phenotypes for *S. aureus* isolates from milk.

Sampling Area	Phenotypes	Number Observed	Percentage (%) Observed
Carletonville (*N* = 7)	PG-AMP-OX-S-K-VA-TEC-E	3	43
	PG-AMP-OX-S-VA-TEC-E	2	29
	PG-AMP-OX-VA-TEC-E	2	29
Potchefstroom (*N* = 4)	PG-AMP-OX-S-VA-TEC-E	2	50
Zeerust (*N* = 10)	PG-AMP-OX-S-VA-TEC-E	3	30
Rustenburg (*N* = 10)	PG-AMP-OX-S-VA-TEC-TE-E-SMX	2	20
	PG-AMP-S-GM-VA-TEC-TE-E	3	30
Stella (*N* = 5)	PG-AMP-OX-S-K-VA-TEC-TE-E-SMX	1	20
Setlagole (*N* = 4)	PG-AMP-OX-S-K-GM-TEC-TE-E-SMX	1	25
Vryburg (*N* = 10)	PG-AMP-OX-S-K-GM-TE-E-SMX	2	20
	PG-AMP-S-K-VA-TEC-TE-E-SMX	3	30
	PG-AP-OX-K-GM-VA-TEC-TE	2	20
Taung (*N* = 5)	PG-AMP-VA-TEC-TE-E	3	60
Disaneng (*N* = 20)	PG-AMP-OX-S-K-GM-VA-TEC-TE-E	2	10
	PG-AMP-OX-S-K-GM-TE-E	3	15
	PG-AMP-OX-S-K-GM-VA-TEC-E	4	20
	PG-AMP-OX-S-K-GM-TEC-E-SMX	3	15
	AMP-OX-S-K-GM	2	10
Mafikeng (*N* = 8)	PG-AMP-VA-TEC-TE-E	2	25
Mabule (*N* = 4)	PG-AMP-S-VA-TEC-TE-E-SMX	1	25
Rooigrond (*N* = 30)	OX-VA-TEC-E	2	7
	VA-TEC-E	3	10
	VA-TEC-TE	2	7
	OX-VA-TEC-TE	2	7
	VA-TEC-TE-E	2	7
Tshidilamolomo (*N* = 2)	PG-AMP-S-VA-TEC	1	50

PG = penicillin G; AMP = ampicillin; OX = oxacillin; S = streptomycin; K = kanamycin; GM = gentamicin; VA = vancomycin; TEC = teicoplanin; TE = tetracycline; E = erythromycin; SMX = sulphamethoxazole.

## 4. Discussion

The primary objective of this study has been to isolate and identify *S. aureus* from milk obtained from supermarkets, shops and farms around the North-West Province, South Africa. The main motivation is that food-borne pathogens, including *S. aureus*, have been isolated from milk and dairy products worldwide [53,54,55,56]. Moreover, a baseline study previously conducted in the area revealed the presence of *S. aureus* in milk collected from a communal farm and two commercial farms [35]. Despite the fact that outbreaks and even sporadic cases of staphylococcal food poisoning have rarely been reported to date in South Africa, the pathogen has been isolated from milk and food products in the area [35,56]. This therefore indicates that there is a possibility of transmitting *S. aureus* and their associated virulence determinants to consumers when unpasteurised milk or its associated products are consumed as is the case in some rural communities in the North West Province of South Africa.

Reasonable attention has been given to pathogenic *S. aureus* strains, especially those equipped with virulence factors even in developed countries that have proper public health facilities [5,57,58]. There is currently little information on the occurrence of this pathogen and its associated virulence determinants in South Africa [35,56]. Data on the occurrence of *S. aureus* and MRSA in South Africa among animals and humans is limited and the determination of its presence in milk may provide an indication of the health risks associated with the consumption of these products and serve as a tool for assessing its public health risks. Previous studies have evaluated the presence of *S. aureus* in milk using conventional microbiology methods [35]. The present study is the first in the area in which a combination of phenotypic, genotypic and proteomic tools were used to concurrently determine the occurrence, antibiotic resistance profiles and virulence gene profiles of *S. aureus* strains isolated from milk samples obtained from retail outlets and farms in the North-West Province, South Africa. Data obtained could provide options for developing *S. aureus* control strategies in the study area.

In the present study, the overall presence of the pathogen was 32.5% among samples screened. However, the proportion of *S. aureus* was higher (75%) in raw milk than in pasteurised milk (29%). This observation was not surprising although it is a cause for concern. The presence of these bacteria, after pasteurisation, can be attributed to inefficacy of the thermal process or post-process contamination. Similar observations have been noticed in which there was a high occurrence of *S. aureus* from raw and pasteurised milk [5,49,59,60]. However, contrary to observations made in some studies, lower proportions of the pathogen have been reported [61,62]. It is therefore suggested that the occurrence of *S. aureus* in milk samples greatly depends on the methods used for isolation, the sample size and geographical region.

In this study, the Pastorex Staph-plus serological assay, amplification of *Staphylococcus* genus specific 16S rRNA gene fragment and the thermo nuclease gene (*nuc*) specific for *S. aureus*, and the MALDI-TOF mass spectrometry were used for positive identification of *S. aureus* strains. Traditionally, bacteria species are identified using phenotypic traits based on results obtained for biochemical tests. Despite the fact that biochemical protocols and assays are constantly being refined, results obtained from these tests are usually not very reliable, and are time consuming [63]. The matrix-assisted laser desorption/ionisation time of light mass spectrometry (MALDI-TOF MS) of intact cells has been reported to be an attractive alternative method that is accurate, rapid, and requires minimal sample preparation time for the correct identification of bacteria isolates.

Different studies have been conducted in which various identification tools were employed for detecting *S. aureus* [62,63,64]. Regardless of the isolation and identification techniques employed, the presence of these pathogens in milk highlights the need for both strict farm management practices and proper sanitary procedures to be implemented during milking operations such as storage, handling and transportation, which are considered to be critical points for *S. aureus* cross contamination. It is therefore suggested that the presence of *S. aureus* in pasteurised milk samples that have not gone past their expiry dates could be attributed to improper handling and holding temperatures during the storage and retail of the products.

Another objective of the study was to determine and compare the antibiotic resistance profiles of *S. aureus* strains isolated from milk samples which may enter the food chain and be transmitted to humans. A motivation for this is that recent studies have revealed an increasing trend towards the occurrence of multiple antibiotic resistant *S. aureus* isolates worldwide [8,35,65]. *S. aureus* isolates that harbour multiple antibiotic resistant traits have been reported to negatively impact on the treatment of staphylococcal infections, especially in immune-compromised individuals, the elderly and young children [66]. In the present study, multiple antibiotic resistant *S. aureus* strains defined as isolates resistant to three or more antibiotics were obtained in a large proportion of milk samples analysed. Development of multiple antibiotic resistance among most of these isolates may be attributed to the acquisition of resistance (R-factor) which is plasmid-mediated [67]. Usually, *S. aureus* is known to contain a number of multiple antibiotic resistant plasmids that may account for the phenotypes observed [67]. In general, a large proportion (60%–100%) of the isolates from different areas and sample types were resistant to penicillin G, ampicillin, oxacillin, streptomycin, vancomycin, tetracycline and erythromycin. Similar patterns of resistance have been reported against *S. aureus* in previous studies [65,68].

The misuse of antibiotics in dairy farms is currently known to be one of the major factors responsible for the emergence of drug resistant bacteria worldwide. Ampicillin and tetracycline are mostly used on dairy cattle farms in the study area and this may account for the resistance levels observed. Furthermore, isolates that are resistant to ampicillin may cross-select for resistance to other β-lactam antibiotics including penicillin and ampicillin resistant isolates may portray β-lactam characteristics and phenotypes. Despite the fact that erythromycin and vancomycin are not used in the area, it has been reported that the use of macrolides tylosin and avoparcin as growth promoters in animals was associated with the emergence of Erythromycin and vancomycin resistance in *S. aureus* [69].

Contrary to the above trends, smaller proportions (22.8%–28%) of the isolates were resistant to Gentamicin, Kanamycin and sulphamethoxazole. Similar observations have been previously reported [63,70]. In addition, these trends may be due to the fact that the antimicrobial agents are rarely or not used at all in animal and human medicine. However, a cause for concern was the detection of oxacillin resistant *S. aureus* isolates in the study using the antibiotic disc susceptibility phenotypic assay despite the fact that the drug is rarely used in both human and animal medicine worldwide. Similar observations have been reported for *S. aureus* from animals, humans, food and water sources [60,62]. Methicillin resistance in staphylococci is due to the presence of the *mecA* gene that encodes for an altered penicillin-binding protein 2a (PBP2a) and therefore, has reduced affinity for β-lactam antibiotics [16]. However, in the present study, only a small proportion (12.1%) of the isolates that exhibited phenotypic resistance to Oxacillin expressed the PBP2a and thus carried the *mecA* gene. Similar observations have previously been reported and it is suggested that in some *S. aureus* strains, phenotypic resistance to oxacillin is not intrinsically mediated by the *mecA* gene [48,71,72], but it is executed through other mechanisms. This non-*mecA* mediated resistance is caused by modifications to the penicillin binding proteins, which results in hyper-production of beta-lactamase or methicillinase enzymes [71]. However, the detection of non-*mecA, S. aureus* strains that are phenotypically resistant to oxacillin and methicillin are known to pose serious health concerns and are of great clinical relevance [72]. Moreover, these non-*mecA S. aureus* strains are still classified as MRSA isolates [72]. Therefore, epidemiological investigations are needed to constantly monitor the antibiotic resistance profile of *S. aureus* strains isolated from different sources in a given area and to implement reliable genetic tracking tools that would facilitate the detection of point source contamination within the food chain. This would greatly reduce the transmission of these pathogens to consumers and reduce the incidence of staphylococcal infections in humans.

A further objective of the study was to determine the presence of staphylococcal virulence genes that include enterotoxin genes, exfoliative genes and collagen adhesin (*cna*) genes using specific PCR analysis. Enterotoxin genes are known to be the major virulence factors of *S. aureus* and therefore responsible for most of the pathogenicity observed in humans, especially when the pathogen is consumed in food products [18]. These toxins are known to modulate immune response through super antigen activity and various diseases caused by strains that produce them [73].

Previous reports designed to detect these toxin genes (enterotoxins, *eta* and *etb* genes) in *S. aureus* revealed great diversity of these genes among isolates from different areas [74,75]. Despite the fact that in the present study 32% of isolates tested possessed the *sec* gene, none of the other virulence determinants were detected. This is not very surprising since the *sec* gene has been previously reported to be the most prevalent virulence factor among *S. aureus* isolates [75,76]. However, the detection of *S. aureus* strains that harbour the *sec* virulence gene indicates that these isolates have the potential to cause either sporadic cases or food borne outbreaks of infections [68].

Similar to previous reports, none of the *eta*, *etb* and *cna* virulence genes were detected in this study [57,77]. In *S. aureus*, the exfoliative toxin genes are associated with bullous impetigo [78] while the collagen adhesin protein (*cna*) gene mediates the binding of clinical isolates to host specific matrix [73]. The *cna* gene is frequently associated with clinical *S. aureus* isolates, although it has also been detected in isolates from both food and environmental sources [75]. Moreover, the *cna* gene product is not easily detected *S. aureus* strains by PCR analysis [79].

In the present study, isolates were assayed for other putative virulence factors including haemolytic activity, the presence of the coagulase enzyme and DNase production that enhance the pathogenic characteristics of *S. aureus* isolates [80]. All the isolates were positive for coagulase activity, which enables them to promote the clotting of fibrin in the blood stream and this is a mechanism by which pathogens protect themselves from destruction during infection [81]. It is therefore suggested that these isolates may present health complications to humans if consumed in contaminated milk or its associated products.

## 5. Conclusions

In the present study, *S. aureus* was successfully isolated from milk samples and a large proportion of *S. aureus* isolates exhibited resistance towards the 11 different antibiotics tested. Thus, it is evident that most of the isolates possessed multiple antibiotic resistance (MAR) attributes. Though the development of antibiotic resistant determinants in *S. aureus* is associated with the uncontrolled usage of antibiotics in human and veterinary medicine, the incidence of drug-resistant *S. aureus* in raw, tank and pasteurised milk samples warrants closer monitoring.

The MAR isolates obtained in this study possessed a number of virulence factors such as haemolysin, coagulase, DNase, catalase, but only few of them carried the enterotoxin gene (*sec*). This supports the notion that though the epidemiological statistics of milk-borne diseases are unavailable, milk products possess pathogenic *S. aureus* strains and may be cause foodborne diseases in consumers. In addition, the *sec* gene was found to be the predominant enterotoxin gene in this genetically diverse South African milk isolates and the presence of this gene is not genotype specific.

## Supplementary Files

Supplementary File 1
