# Supplementary material for: Detection of Antibiotic Resistant Staphylococcus aureus from Milk: A Public Health Implication
Source: Int J Environ Res Public Health. 2015 Aug 25;12(9):10254–75. doi: 10.3390/ijerph120910254 (PMC4586610; doi:10.3390/ijerph120910254)
Supplement: Supplementary File 1 [file ijerph-12-10254-s001.pdf]

Supplementary Information

Detection of Antibiotic Resistant *Staphylococcus aureus* from Milk: A Public Health Implication

---

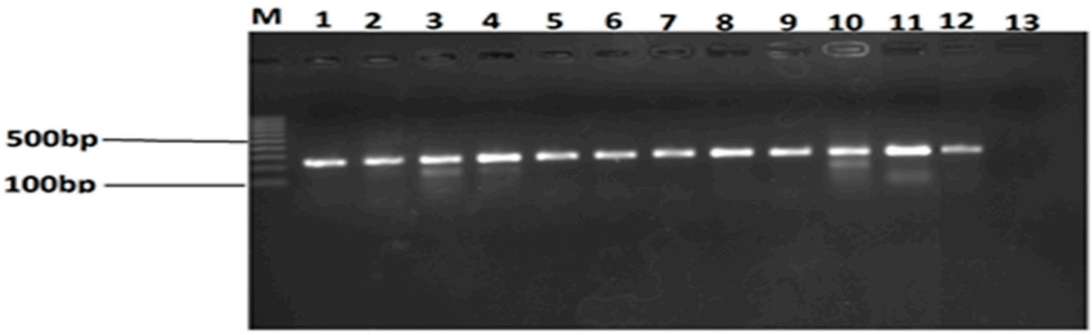

Figure S1. Agarose gel electrophoresis analysis for the 16rRNA gene in *S. aureus* isolates. Lane M = 100bp DNA maker, Lanes 1–11 = *S. aureus* isolates, lane 12 = *S. aureus* 25923 (positive control), lane 13 = negative control.

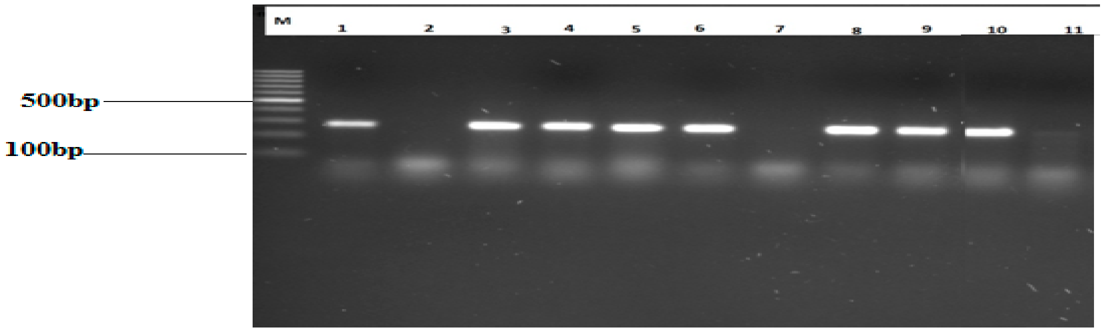

Figure S2. Agarose gel electrophoresis analysis for the *nuc* gene in *S. aureus* isolates. Lane M = 100bp DNA maker; Lanes 1, 3, 4, 5, 6, 8 and 9 = *S. aureus* isolates; lanes 2 and 7 = nuc negative isolates; lane 10 = *S. aureus* 25923 (positive control); lane 11 = negative control.

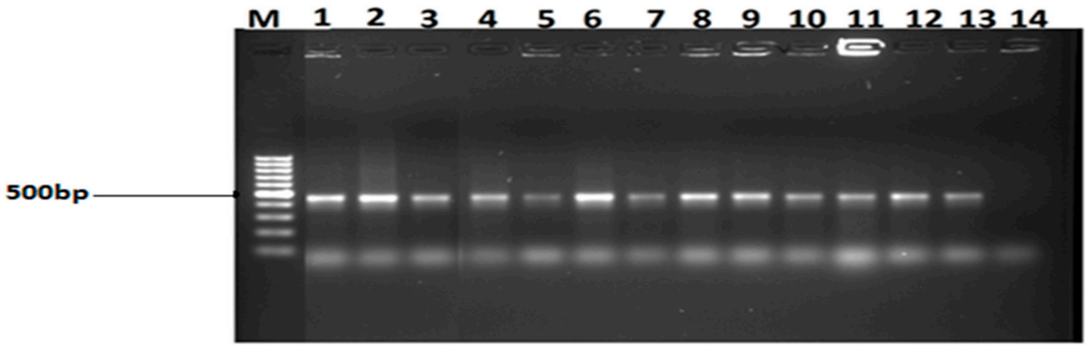

Figure S3. Agarose gel electrophoresis analysis for the *sec* gene in *S. aureus* isolates. Lane M = 100bp DNA maker, Lanes 1–13 = *S. aureus* isolates, lane 14 = negative control.

Isolate Number	Score Value	Identity based on MALDI-TOF MS analysis<br>Best Match	Score Value	Identity based on MALDI-TOF MS analysis<br>Second Best Match
**1RO3**	2.234	*Staphylococcus aureus*	2.073	*Staphylococcus aureus*

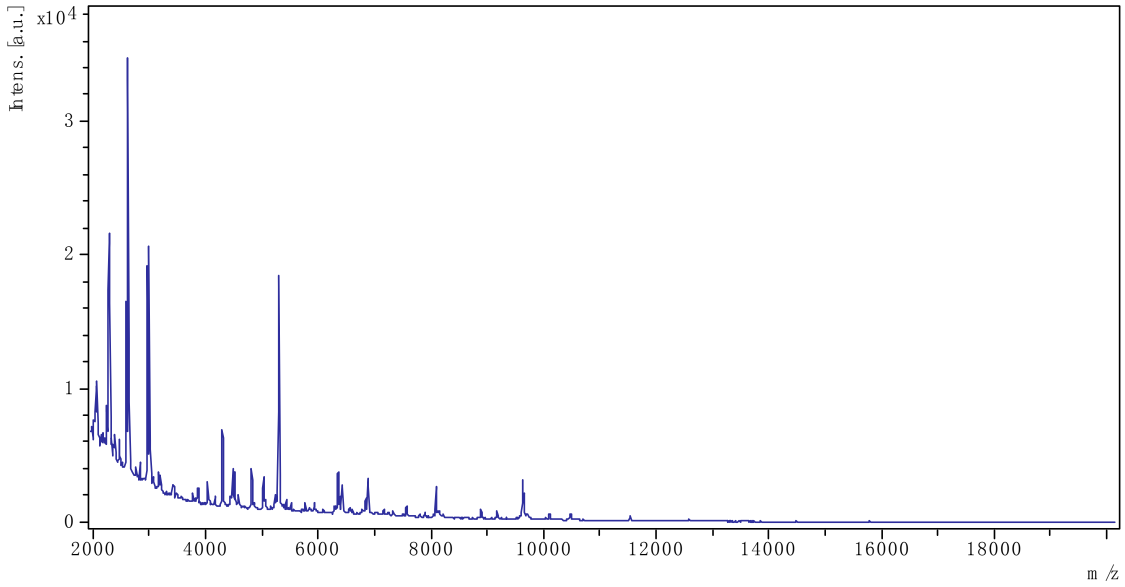

**1RO4**	2.097	*Staphylococcus aureus*	1.936	*Staphylococcus aureus*

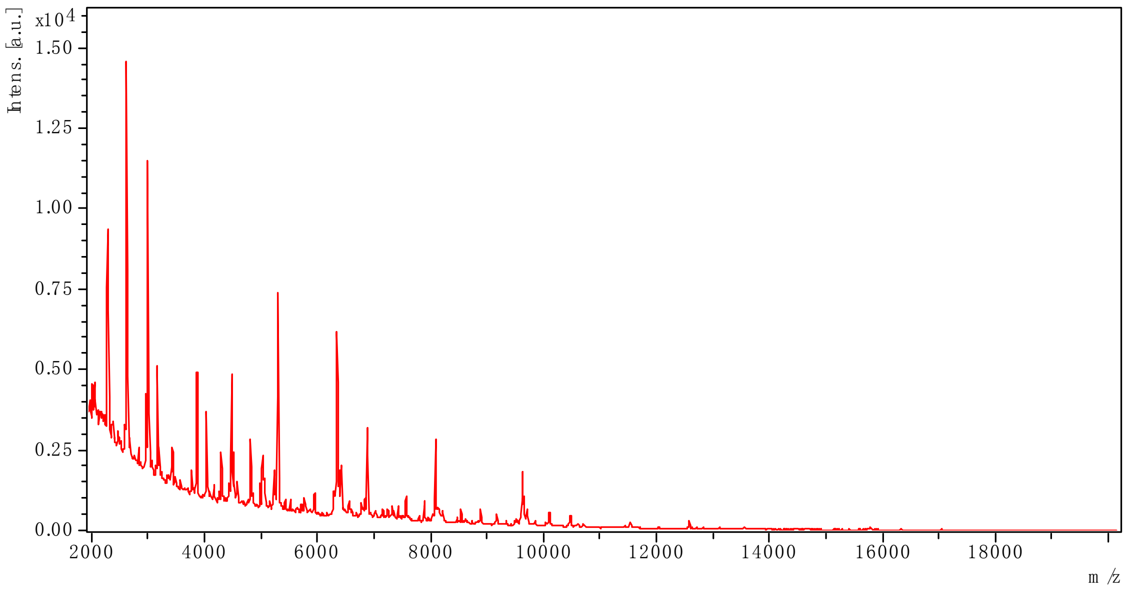

Figure S4. A representative mass spectral profiles of *S. aureus* isolated from milk obtained from Rooigrond.

© 2015 by the authors; licensee MDPI, Basel, Switzerland. This article is an open access article distributed under the terms and conditions of the Creative Commons Attribution license (http://creativecommons.org/licenses/by/4.0/).
